# Impact of preoperative geriatric screening and comorbidity assessment in patients with vulvar and vaginal cancer

**DOI:** 10.1007/s00432-025-06378-5

**Published:** 2025-12-08

**Authors:** Valerie Catherine Linz, Emma Liebau, Markus Schepers, Katharina Gillen, Marco Johannes Battista, Michael Mohr, Mona Wanda Schmidt, Marcus Schmidt, Annette Hasenburg

**Affiliations:** 1 Department of Obstetrics and Gynecology, University Medical Center of the Johannes Gutenberg University Mainz, Langenbeckstraße 1, 55131 Mainz, Germany; 2 Institute of Medical Biostatistics, Epidemiology and Informatics (IMBEI), University Medical Center of Johannes Gutenberg University Mainz, Langenbeckstraße 1, 55131 Mainz, Germany; 3 Department of Gynecology, Diakonie Hospital Jung-Stilling Siegen, Wichernstraße 40, 57074 Siegen, Germany; 4 Geriatric Department, University Medical Center of the Johannes Gutenberg University Mainz, Langenbeckstraße 1, 55131 Mainz, Germany

**Keywords:** Vulvar cancer, Vaginal cancer, Frailty, Pre-screening, G8-screening tool, Geriatric assessment

## Abstract

**Purpose:**

Patients with vulvar (VC) and vaginal (VaC) cancer are often frail and should be prescreened before a time-consuming comprehensive geriatric assessment (CGA). This study assessed the impact of the preoperatively determined frailty status with the G8 geriatric screening tool (G8) and comorbidity assessment on the outcome of patients with VC/VaC.

**Methods:**

We conducted an observational study with prospective data collection of patients aged ≥ 60 undergoing surgery for VC and VAC from 05/2020 to 01/2025. Patients were assessed with the G8 tool, age-adjusted Charlson-Comorbidity Index and the Lee-index. Positive G8 results led to CGA-based testing and, if indicated, geriatric consultation. Cox regression, Kaplan–Meier curves and propensity score matching (PSM) were used to analyze the predictive validity of the G8.

**Results:**

41 patients were screened. 26 patients were included with a mean follow-up of 23 months. 10 patients were considered G8 positive (G8 ≤ 14 points). Median age was 74.5 (interquartile range: 66–81.3) years. The G8 positive cohort was older, had more comorbidities and had higher ECOG and ASA performance status than the G8 negative cohort. 20% of the G8 positive patients did not receive standard surgical therapy and only one in five patients underwent standard adjuvant radio-/chemotherapy. The univariate Cox-model for overall survival (OS) for G8 positivity had a hazard ratio of 5.65 with 95% CI [1.14–28.1] and a significantly lower 2-year OS (40.0 vs. 85.1%), supported by PSM adjusted for residual confounding (*p* = 0.017).

**Conclusions:**

The G8 can be easily implemented in the clinical routine to identify VC and VaC patients with a reduced 2-year OS who may benefit from CGA.

**Supplementary Information:**

The online version contains supplementary material available at 10.1007/s00432-025-06378-5.

## Background

Vulvar (VC) and vaginal cancers (VaC) are rare but significant gynecological malignancies. In 2022, 47.342 new cases with VC and 18.800 with VaC were diagnosed worldwide (Bray et al. 2024). Surgery is the primary treatment for early-stage VC and VaC, aiming for complete tumor resection with clear margins. Despite becoming less radical in the past decades, surgical therapy still causes a high rate of complications (Rahm et al. 2022) and anatomical changes that can significantly deteriorate the patient’s quality of life (Gunther et al. 2014). According to the European Society of Gynaecological Oncology (ESGO) guidelines for the management of VC, preoperative work-up also includes a “frailty assessment” (Oonk et al. 2023). However, specifications are lacking, and the optimal way to integrate geriatric expertise into the surgical setting has yet to be defined. Frailty plays a significant role in the prognosis and treatment of VC and VaC patients, particularly in predominantly affected older women (Gans et al. 2023). In general, frailty seems to be a key predictor of mortality in patients with gynecological cancer, regardless of their chronological age (Mullen et al. 2021). Frailty gradually arises as the body’s systems weaken, leading to reduced strength and resilience and a higher risk of falls, hospitalizations, and serious health complications (Clegg et al. 2013; Hoogendijk et al. 2019). Frail cancer patients are identified through a comprehensive geriatric assessment (CGA) to help shape cancer treatment decisions and tackle any challenges through the correct interventions, support, and referrals. GA should focus on key areas affecting older cancer patients, including physical and mental health, emotional well-being, comorbidities, medication, nutrition, and social support. All cancer patients older than 65 years should receive a GA before systematic therapy according to the updated ASCO (American Society of Clinical Oncology) guidelines (Dale et al. 2023). However, only 52% of the ASCO members, when surveyed in 2019, reported being aware of this guideline (Dale et al. 2021). Despite the growing evidence that GA can enhance treatment outcomes by reducing chemotherapy-related toxicity, lowering complication rates after surgery, and improving both functional status and quality of life (Rostoft et al. 2021; Hamaker et al. 2022), GA is often not implemented in clinical routine due to limited staff capacity, competing demands of time, provider inexperience, and misaligned institutional priorities (Seaman et al. 2023). Prescreening is commonly employed to identify patients who are fit for standard treatment, while those requiring a full GA can be further assessed. The G8 is one of the few prescreening methods designed specifically for the assessment of frailty in patients with cancer and planned systematic therapy by Bellera et al. (2012). However, Bellera et al. (2012) underlined that the G8 “is not aimed at replacing the expertise of geriatricians for the diagnosis of frailty”. The G8 is also an established simple and useful screening method for patients undergoing cancer surgery, e.g. for assessing the risk of postoperative complications (Horiuchi et al. 2024). Moreover, positive G8 scores were associated with reduced OS in the ELCAPA cohort with older cancer patients, regardless of their metastatic status or tumor site (Martinez-Tapia et al. 2017). The G8 consists of 8 questions and can be implemented in the clinical practice without the need for geriatric counseling. Its areas of focus encompass age, nutrition, mobility, neuropsychological issues, medication, and self-reported health. In a meta-analysis by Hamaker et al. (2012), the G8 had one of the highest sensitivity for frailty on comprehensive GA with a median sensitivity of 87% (range 77–92%), but a reduced specificity with a median of 61% (range 39–75%).

According to the current literature, little is known about VC/VaC and frailty, especially in a prospective setting. Most of the studies included all gynecological tumors and not exclusively patients with VC and VaC. Therefore, the aim of our study was to explore the association between preoperative geriatric screening, with a particular focus on the G8, comorbidity assessment, and patient outcomes.

Given the limited sample size, analyses of survival outcomes were considered exploratory, while greater emphasis was placed on short-term perioperative endpoints and treatment allocation by frailty status, which provide clinically relevant insights for surgical decision-making. According to our institutional perioperative risk management protocol, patients with a BMI > 35 kg/m^2^ were considered at increased perioperative risk. Therefore, a BMI > 35 kg/m^2^ was defined as an inclusion criterion to ensure representation of patients with elevated frailty-related risk profiles.

## Material and methods

### Data collection and frailty assessment tools

All gynecologic oncological patients aged 60 years and older and/or with a body mass index (BMI) > 35 kg/m^2^; reflecting the institution’s predefined threshold for severe obesity and increased perioperative risk; and/or who seemed subjectively frail were systematically screened in a two-step frailty assessment before tumor surgery at the University Medical Center Mainz from May 2020 till January 2025 within the ongoing Frail-B study (https://www.drks.de/search/de/trial/DRKS00032361/details).

Precursor lesions, in this case vulvar intraepithelial neoplasia (VIN), were excluded, as well as patients with metastatic or inoperable cancer, vulvar melanoma or missing follow-up (FU). The first screening assessment encompassed the G8, Lee-index, standard blood parameters and hand grip strength (since 2023). If assessed as G8 positive, patients were screened for falls, and underwent the following tests with regard to CGA: History of falls in the last six months, Mini-Cog, Barthel Index, Geriatric Depression Scale (GDS), and the Timed Up and Go (TUG) test. If one of the second assessment tests showed indications of frailty, geriatric counseling was included.

The primary endpoint was the correlation between frailty status and postoperative complications in patients with gynecologic tumors. Secondary endpoints included survival outcomes in relation to preoperative screening tools. The following baseline data were collected from patient files: demographics, BMI, tumor characteristics, perioperative complications, laboratory results and type of treatment. The scoring system for the G8 ranges from 0 points (heavily impaired—G8 positive) to 17 points (not impaired at all—G8 negative), with a cutoff value of ≤ 14 points (Bellera et al. 2012). After scoring each comorbidity, the patient’s 10-year survival rate was calculated according to the age-adjusted CCI (aCCI) with an online tool (https://www.mdcalc.com/calc/3917/charlson-comorbidity-index-cci) (Charlson et al. 1987; Quan et al. 2011; Radovanovic et al. 2014). For each patient, “localized solid tumor” was excluded (+ 2 points). If a patient had suffered from another cancer before VC or VaC, the diagnosis was included (e.g. breast cancer). Lee developed a 4-year mortality (Lee et al. 2006; Yourman et al. 2012) and was calculated online with https://eprognosis.ucsf.edu/leeschonberg.php. Lee frailty was defined as a Lee-index ≥ 8 points and a 4-year mortality rate of ≥ 20% without including the current cancer diagnosis (Anic et al. 2022). Ethical approval for this study was obtained from the State Medical Association of Rhineland-Palatinate (ID: 16691).

### Statistics

Descriptive statistical analysis was performed with IBM SPSS Statistics 29.0.2.0 and the propensity-score matching with R version 4.2.3 (Denz et al. 2023; Team 2023). Patients’ characteristics were given as absolute and relative frequencies, as mean ± standard deviation (SD), or median with interquartile ranges (IQR). Normal distribution was tested with the Shapiro–Wilk test, followed by either a Mann–Whitney U test or a t-test to assess significant differences. Categorical variable distributions were compared using the Chi-square or Fisher’s exact test. The Cox proportional hazard regression model was used to evaluate the prognostic impact of established risk factors (e.g. age, ECOG PS, FIGO stage). Univariate Cox regression was performed for each variable, and those with *p* < 0.05 were included in the multivariable model using backward elimination. Given the small subgroup sizes (e.g., FIGO III, ECOG ≥ 2), hazard-ratio estimates are imprecise; analyses should be interpreted as exploratory. Kaplan–Meier estimates described recurrence-free survival (RFS) and OS at 2 years. Time points were defined from surgery to death (or recurrence), or last FU. Patients alive (or without recurrence), or with incomplete data, were censored. RFS included loco-regional recurrences, distant metastases, and death as events. Hazard ratios (HR) with 95% confidence intervals (CI) were calculated in the Cox regression model, with the Log-Rank test used to compare curves. All tests were two-sided, and *p* < 0.05 was considered significant. No correction for multiple testing was applied, so the results are exploratory. To account for the imbalance between the treatment and control group in terms of potential confounders, a propensity score-matched (PSM) sample was obtained, using the “full” method, as it gave the strongest reduction in standardized mean difference between the cohorts. The propensity score was calculated using the covariates aCCI, ECOG PS (at surgery) and Lee-Index with a LASSO logistic regression which selected these covariates from: age, BMI, FIGO stage, aCCI, ECOG PS (at surgery) and recurrence surgery. Kaplan–Meier survival curves were generated to compare outcomes between G8 positive and G8 negative cohorts within the matched sample.

The manuscript followed the STROBE (Strengthening the Reporting of Observational Studies in Epidemiology) guidelines (Vandenbroucke et al. 2007).

## Results

### Patients’ characteristics

41 patients with suspected VC and VaC were screened, 26 patients were included in the final analysis of whom 2 patients had VaC. 15 patients were excluded due to having only precursor lesions, melanoma, only diagnostic or palliative surgeries or missing FU. 10 of all patients were G8 positive (38.5%). 20 patients underwent surgery at initial diagnosis, 6 patients received surgical treatment for loco-regional recurrence. Most of the patients had early VC and VaC cancer with stage FIGO I (73.1%). Median age was 74.5 (IQR 66–81.3) years. On average, the G8 positive cohort was 8 years older than the G8 negative cohort (*p* = 0.016). Most of the patients were evaluated with an ECOG PS 0–1 (77%) at surgery. The G8 positive cohort had higher ECOG PS (ECOG PS ≥ 2: 60% vs. 0%; *p* = 0.004) and ASA PS (ASA 3: 80% vs. 12.5%; *p* ≤ 0.001). G8 positive patients were on significantly more medication (≥ 5) before surgery (*p* = 0.001). Regarding the aCCI and comorbidities, the G8 positive cohort presented with a higher risk of mortality than the G8 negative cohort (80% vs. 37.5%; *p* = 0.034) and 50% of the G8 positive cohort had an estimated 10-year-survival of 2%. The risk of 4-year mortality according to Lee was also higher in the G8 positive cohort (*p* = 0.003). All parameters are listed in Table 1.

**Table 1:** Baseline characteristics

Parameter	All VC/VaC patients n = 26 (%)	G8 negative n = 16 (%)	G8 positive N = 10 (%)	*p* value
VC	24 (92.3)	14 (87.5)	10 (100)	
VaC	2 (7.7)	2 (12.5)	–	
Initial diagnosis	20 (76.9)	13 (81.3)	7 (70)	0.158
Loco-regional recurrence	6 (23.1)	3 (18.8)	3 (30)	
First recurrence	2 (7.7)	–	- 2 (20)	
Second recurrence	2 (7.7)	2 (12.5)	–	
Third recurrence	1 (3.8)	1 (6.3)	–	
Forth recurrence	1 (3.8)	–	1 (10)	
FIGO stage				0.61
FIGO I	19 (73.1)	12 (75)	7 (70)	
FIGO II	1 (3.8)	1 (6.3)	–	
FIGO III	6 (23.1)	3 (18.8)	3 (30)	
FIGO IV	–	–	–	
Histological grading				0.367
G1	3 (11.5)	1 (6.3)	2 (20)	
G2	16 (61.5)	9 (56.3)	7 (70)	
G3	5 (19.2)	4 (25)	1 (10)	
unkown	2 (7.7)	2 (12.5)		
Median chronological age [years] (IQR)	74.5 (66–81.3)	70.5 (64.3–76.8)	81 (73.8–84)	**0.016**
Mean chronological age [years] (± SD)	74 (± 8.8)	71 (± 8.5)	79 (± 7.3)	
Mean Body Mass Index [kg/m^2^] (± SD)	28.3 (± 7.2)	27.2 (5.5)	30.2(± 9.4)	0.29
ECOG PS				**0.004**
0	10 (38.5)	9 (56.3)	1 (10)	
1	10 (38.5)	7 (43.8)	3 (30)	
2	3 (11.5)	–	3(30)	
3	3 (11.5)	–	3 (30)	
ASA PS				**< 0.001**
0	–	–	–	
1	–	–	–	
2	15 (57.7)	14 (87.5)	1 (10)	
3	10 (38.5)	2 (12.5)	8 (80)	
unkown	1 (3.8)	–	1 (10)	
Polypharmacy ≥ 5	8 (30.8)	1 (6.3)	7 (70)	**0.001**
aCCI*				**0.034**
Low risk (0–1 points)	–	–	–	
Intermediate risk (2–3 points)	12 (46.2)	10 (62.5)	2 (20)	
High risk (≥ 4 points)	14 (53.8)	6 (37.5)	8 (80)	
aCCI*				
10-year survival				
90%	4 (15.4)	3 (18.8)	1 (10)	
77%	8 (30.8)	7 (43.8)	1 (10)	
53%	5 (19.2)	3 (18.8)	2 (20)	
21%	4 (15.4)	3 (18.8)	1 (10)	
2%	5 (19.2)	–	5 (50)	
Risk of 4-year mortality according to Lee *				**0.003**
< 5%	14 (53.8)	13 (81.3)	1 (10)	
6–9%	8 (30.8)	3 (18.8)	5 (50)	
15–20%	2 (7.7)		2 (20)	
20–28%	2 (7.7)		2 (20)	
44–45%	–	–	–	
59%	–	–	–	
64%	–			
Lee*-frail (≥ 8points) and 4-year mortality ≥ 20%	3 (11.5)	–	3 (30)	**0.046**

*aCCI* age-adjusted Charlson comorbidity index, *ASA PS* American Society of Anesthesiologists Physical Status, *ECOG PS* Eastern Cooperative Oncology Group Performance Status, *FIGO* International Federation of Gynecology and Obstetrics, *HPV* human papillomavirus, *IQR* interquartile range, *SD* standard deviation, *VaC* vaginal cancer, *VC* vulvar cancer. Bold figures indicate statistical significance

*aCCI and Lee-Index were calculated without the current vulvar/vaginal cancer diagnosis to assess the patient’s general life expectancy/ mortality rate

Handgrip strength measurement results obtained since 2023, comprising data from six patients, are shown in Supplementary Table 1.

### Treatment characteristics

Regarding surgical treatment, there was no difference in the extent of the surgery between the two cohorts. All surgical details are listed in Table 2. 80.8% of the patients received radical local excision, and 61.5% had inguinal sentinel lymph node staging. Seven patients underwent inguinofemoral lymphadenectomy. Both VaC patients had partial colpectomy. The mean minimum tumor-free surgical margins were 4.85 mm. 20% of the G8 positive cohort did not receive standard pelvic lymph node staging, otherwise all patients received standard surgical therapy. The G8 positive cohort had significantly more de-escalated adjuvant therapy than the G8 negative cohort. Only one in five G8 positive patients underwent the recommended adjuvant radio/chemotherapy. The other four patients did not receive adjuvant therapy due to their frailty. One G8 negative patient refused the recommended radiation therapy of the groins due to different reasons. Three patients were assessed as Lee-frail. All three Lee-frail patients belonged to the G8 positive cohort. Two in three Lee-frail patients did not receive standard therapy.

**Table 2:** Treatment characteristics

Parameter	All VC/VaC patients n = 26 (%)	G8 negative n = 16 (%)	G8 positive N = 10 (%)	*p* value
Surgical treatment				*p* > 0.05
Radical local excision	21 (80.8)	13 (81.3)	8 (80)	
SN without IFL or LN debulking	16 (61.5)	10 (62.5)	6 (60)	
IFL or LN debulking	7 (26.9)	3 (18.8)	4 (40)	
Pelvic LN staging	4 (15.4)	4 (25)	–	
TLH	2 (7.7)	2 (12.5)	–	
Partial colpectomy (vaginal cancer)	2 (7.7)	2 (12.5)	–	
Mean minimum tumor-free surgical margin in mm	4.85 (± 3.2)	5 (± 3.3)	4.5 (± 3.2)	0.665
Mean operating time [minutes] (± SD)	140.8 (± 94.2)	158.4 (± 102.2)	112.6 (± 74.1)	0.24
Standard surgical therapy	24 (92.3)	16 (100)	8 (80)	0.138
No pelvic LN staging	2 (7.7)	–	2 (20)	
Standard adjuvant therapy	22 (84.6)	16 (100)	6 (60)	**0.014**
No further therapy necessary	16 (61.5)	11 (68.8)	5 (50)	
Radio-/chemotherapy	6 (23.1)	5 (31.3)	1 (10)	
No radio-/chemotherapy due to frailty	4 (15.4)		4 (40)	
30-days-postoperative complications				
Wound dehiscence	7 (26.9)	4 (25)	3 (30)	
Wound infection	6 (23.1)	4 (25)	2 (20	0.562
Serom	9 (34.6)	4 (25)	5 (50)	
Cardiac arrest/infarction	–	–	–	
Pulmonary	–	–	–	
Nephrological	–	–	–	
Thromboembolic	–	–	–	
Blood transfusion	1 (3.8)	–	1 (10)	
Clavien-Dindo-Classification				0.303
0	9 (34.6)	5 (31.3)	4 (40)	
I	6 (23.1)	5 (31.3)	1 (10)	
II	5 (19.2)	3 (18.8)	2 (20)	
IIIa	4 (15.4)	3 (18.8)	1 (10)	
IIIb	2 (7.7)	–	2 (20)	
IV	–	–	–	
V	–	–	–	
Median length of hospitalization [days] (IQR)	7 (5–11.25)	6.5 (5–11.75)	7.5 (5.8–14)	0.53
Mean length of hospitalization [days] (± SD)	10.3 (± 11.6)	8.1 (± 4.6)	14 (± 17.8)	
Revision	2 (7.7)	–	2 (20)	0.138
Readmission rate	0	–	–	
Median follow-up period [months] (IQR)	22 (10.3–34)	27.5 (12–42.5)	20.5 (5.3–27.3)	0.086
Mean follow-up period [months] (± SD)	23.0 (± 15.3)	26.8 (± 16.4)	17 (± 11.7)	
Recurrence during follow-up	8 (30.8)	6 (37.5)	2 (20)	0.321
Death	8 (30.8)	2 (12.5)	6 (60)	**0.017**
Cancer-related	3 (11.5)	2 (12.5)	1 (10)	
Treamtent-related	1 (3.8)	–	1 (10)	
Other causes	1 (3.8)	–	1 (10)	
unkown	3 (11.5)	–	3 (30)	

*IFL* inguinofemoral lymphadenectomy, *IQR* interquartile range, *LN* lymph node, *SD* standard deviation, *SN* sentinel node procedure, *TLH* total laparoscopic hysterectomy, *VaC* vaginal cancer, *VC* vulvar cancer. Bold figures indicate statistical significance

The standard laboratory results were generally within normal limits.

Regarding surgical complications, mean length of hospital stay and the revision rate, the G8 positive cohort did not show inferior results compared to the G8 negative patients. However, significantly more G8 positive patients died during the mean FU of 23 months (60% vs. 12.5%), see Table 2.

Of the G8 positive patients, eight underwent the second screening algorithm, while two declined participation. The results are descriptive and listed in Supplementary Table 2. Five were scheduled for geriatric counseling, of which only one session was completed. In this case, the alterations observed in the CGA were considered possibly indicative of early-stage dementia. Recommendations included optimizing fluid intake and enhancing home care support. No treatment de-escalation was advised. The remaining four counseling sessions could not be conducted due to difficulties in scheduling appointments prior to surgery and staff shortages. Two of the five patients scheduled for the cGA had passed away within eight months after surgery.

### Survival analysis and PSM

The 2-year OS, estimated with the Kaplan–Meier curves, was significantly reduced in the G8 positive cohort (40% vs. 85.1%; *p* = 0.019). RFS did not differ between the G8 positive and G8 negative cohort (*p* = 0.510).

In the uni- and multivariate Cox-regression analysis for RFS including age at surgery, ECOG PS, FIGO stage, aCCI and G8, only age at surgery retained its significant influence on RFS, see Table 3. Even in a propensity-score-matched data set based on age (“nearest” method with caliper 0.2, leading to a sample size of n = 12), age remained significant in a univariate Cox-regression for RFS (HR 1.17, *p* = 0.01). Furthermore, in a propensity score matching based on comorbidity, ECOG PS and Lee-Index (n = 20), age was significant in a Cox-regression for RFS (HR = 1.1, *p* = 0.04).

**Table 3:** Cox-regression analysis

	Univariate RFS			Multivariate RFS			Univariate OS			Multivariate OS		
	HR	CI [95%]	*p* value	HR	CI [95%]	*p* value	HR	CI [95%]	*p* value	HR	CI [95%]	*p* value
Age at surgery	1.11	1.02–1.20	**0.011**	1.09	1.02–1.19	**0.011**	1.24	1.06–1.44	**0.006**	1.16	0.99–1.37	0.068
ECOG PS (0/1 vs. ≥ 2)	3.21	1.04–9.9	**0.042**	1.95	0.58–6.54	0.282	9.51	2.24–40.45	**0.002**	11.03	1.06–114.91	**0.045**
FIGO stage Local (I–II) vs. advanced (III)	3.04	0.95–9.7	0.061				5.03	1.23–20.61	**0.025**	12.78	1.36–119.78	**0.026**
aCCI (≤ 3 vs. ≥ 4 points)	1.82	0.6–5.6	0.293				8.38	1.03–68.6	**0.047**	5.6	0.23–135.36	0.289
G8 negative vs. G8 positive	1.43	0.48–4.27	0.521				5.51	1.11- 27.34	**0.037**	0.22	0.12–4.18	0.316

Each factor was evaluated in a separate univariate Cox proportional hazards regression model, stratified by cohort (referent vs. variable X) if possible. Bold figures indicate statistical significance

*aCCI* age-adjusted Charlson comorbidity index, *HR* hazard ratio, *CI [95%]* 95%-confidence interval, *FIGO* International Federation of Gynecology and Obstetrics, *ECOG PS* Eastern Cooperative Oncology Group Performance Status

For OS, only higher ECOG PS (≥ 2) and higher FIGO stages (stage III) had a significantly negative impact. Due to the small sample size and low number of events, no further parameters could be included in the multivariate Cox regression analysis, e.g. ASA PS, or surgical treatment variables.

**Fig. 1:**
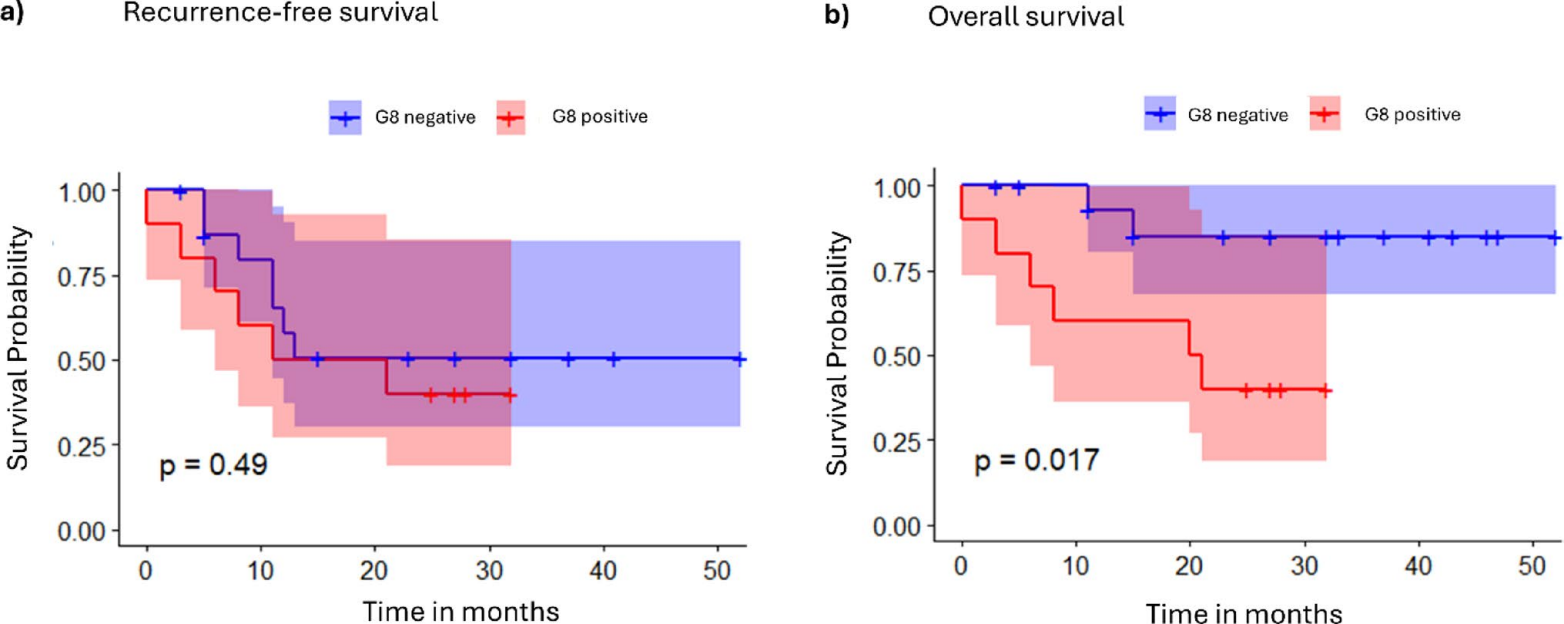
Propensity score matched survival estimates. **a** Adjusted recurrence-free survival: G8 negative vs G8 positive. **b** Adjusted overall survival: G8 negative vs G8 positive

Regarding PSM, the univariate Cox-model for RFS for G8 positivity had a hazard ratio (HR) of 1.45 with 95% CI [0.49–4.31], i.e. a lower but non-significant survival. However, there was significant residual confounding in ECOG PS. The univariate Cox-model for OS for G8-positivity had a HR of 5.65 with 95% CI [1.14–28.1], i.e. a significantly lower OS (*p* = 0.017). There was residual confounding in comorbidity, ECOG PS and Lee-index. The propensity score matched survival curves are shown in Fig. 1. In both cases, the effect direction of the HR changed in the model adjusted for the residual confounding due to the small sample size. Since there was only one control unit with at least a moderate propensity score; the propensity scores of the controls were lower than those of the treated.

## Discussion

Our study demonstrates that G8 positive VC and VaC patients had a significantly reduced OS. Notably, 20% of the G8 positive patients did not receive standard surgical therapy, and only one in five patients underwent standard adjuvant radio-/chemotherapy. These findings highlight the need for an interdisciplinary, individualized therapeutic approach. The G8 may serve as an effective, preoperative screening tool to identify VC and VaC patients at risk which should be targeted upfront before initiating treatment.

Older VC patients are often diagnosed at more advanced tumor stages, with a higher frequency of HPV-negative tumors, leading to an increased risk of RFS and a reduced 2-year disease-free survival (DFS) (Prieske et al. 2021). In our study, advanced age was associated with negative RFS, while higher ECOG PS correlated with reduced OS. These findings align with the AGO-CaRE-1 study, which identified age, ECOG PS, R1 resection, tumor stage, grading, and (chemo)radiation as key prognostic factors for recurrence and survival (Prieske et al. 2021). A recent multicenter study by the Francogyn group further supported the association between advanced age, larger tumor size, and increased surgical complexity in VC patients, elevating complication risk and reducing the oncological outcome (Raimond et al. 2024).

Frailty plays a crucial role in treatment decisions. A retrospective cohort study by Gans et al. found frailty in 45.6% of VC patients aged 70 or older, with oncological treatment being de-escalated in half of these cases (Gans et al. 2023). When a geriatrician was involved in decision-making, nearly two-thirds of frail patients had treatment de-escalation, yet survival outcomes remained unaffected. However, cohort heterogeneity, particularly the inclusion of patients with FIGO stage IV disease, complicates broad conclusions regarding treatment adjustments. No impact of G8 positivity on the standard oncological treatment was found (n = 27). Similarly, Reiser et al. reported that even among patients with gynecological malignancies aged 80 and older, including 37 VC patients, standard therapy was feasible irrespective of frailty, although frail patients exhibited shorter progression-free and OS. In their study, the frailty index was retrospectively calculated with 31 items (Reiser et al. 2021). In general, tailoring oncological care, e.g. de-escalating chemotherapy, does not compromise survival (Li et al. 2021; Mohile et al. 2021). In our study, no therapeutic adjustments were made following geriatric counseling. However, only one out of five scheduled appointments was conducted, primarily due to limited staff availability and challenges in coordinating the appointments. Two of these five G8 positive patients died within eight months after surgery. This raises the question whether these patients would have benefited from that planned geriatric consultation and a potential treatment de-escalation. Due to the limited sample size, we were unable to evaluate the impact of de-escalated therapy or a CGA on the oncological outcome. It remains unclear whether the reduced OS is attributable to the de-escalated treatment itself or primarily influenced by the patients’ overall life expectancy. This analysis is planned for future studies.

Frailty assessment tools can predict treatment outcomes and postoperative complications. The Rockwood Accumulation of Deficits Frailty Index (DAFI) has demonstrated prognostic value in patients with endometrial cancer, VC, and VaC and those with loco-regional disease only. Mullen et al. analyzed 186 VC and VaC patients from the SEER-MHOS database from 1998 to 2015 (Mullen et al. 2021). Frailty can also be linked to adverse outcomes. An NSQIP database analysis showed that nearly 25% of women undergoing radical vulvectomy were considered frail according to the 5-factor modified frailty index (mFI-5). Frailty was associated with increased postoperative complications, especially in women undergoing bilateral inguinofemoral lymphadenectomy (Levine et al. 2023). However, Delli Carpini et al. found no statistically significant association between mFI-5 and 30-day postoperative complications at the logistic regression, possibly due to parameter overlap with the aCCI (4/5 parameters) (Delli Carpini et al. 2024). Interestingly, our study found no increased surgical complications among G8 positive patients, potentially due to the limited sample size. Nevertheless, aCCI showed predictive value for prognosis and complications in surgically treated VC patients (Di Donato et al. 2019). ACCI and BMI also predicted the risk of complications for patients undergoing surgery for VC in a recently published retrospective study including 225 VC patients from two centers from 2018 to 2023 (Delli Carpini et al. 2024). Preoperative assessment of complication risk aids in surgical planning and enables a customized peri- and postoperative management strategy.

### Strengths and limitations

To our knowledge, this is the first study with prospective data collection investigating frailty and oncological outcomes in VC and VaC patients that provides novel data on the prognostic relevance of the G8. Unlike previous studies, our research was based on recent data from the last 5 years. However, VC and VaC are rare diseases, and existing studies on frailty in this context remain scarce. Despite our efforts to minimize bias by excluding patients with metastatic disease, melanoma, or missing FU data, our study cohort was small and heterogeneous. Its non-interventional observational design and small sample size limit the ability to draw definitive conclusions regarding long-term oncologic outcomes. Therefore, we emphasize that the survival analyses should be viewed as exploratory, and that our results primarily demonstrate the feasibility and potential clinical relevance of integrating geriatric screening into preoperative workflows. Patients included both newly diagnosed and recurrent cases, which could influence outcomes. Although we applied Cox regression analysis and PSM to strengthen our findings, residual confounding due to limited statistical power was still a problem.

### Clinical implications and conclusion

The integration of CGA into clinical practice remains a challenge due to time constraints and limited staff capacity. However, the G8 screening tool offers a practical alternative for identifying potentially frail patients at risk of de-escalated therapy and poor oncological outcome, even in settings where full GA is not feasible. Our findings suggest that preoperative geriatric screening, including the G8, and comorbidity assessment may help identify patients with VC and VaC at higher risk of adverse outcomes and could inform timely risk stratification and treatment adaptation. G8 positive patients should be identified preoperatively and targeted upfront before initiating treatment. However, given the modest sample size and small subgroups, these results should be interpreted as hypothesis-generating, and confirmation in lager, multi-center studies is required before any practice recommendations. In general, the precise implementation of a potential prehabilitation for frail patients or therapy adjustments remain an area of ongoing research.

## Supplementary Information

Below is the link to the electronic supplementary material.

Supplementary Material 1

## Data Availability

The data supporting the findings of this study are not publicly available, as they contain information that could compromise the privacy of the research participants. Data are available from the corresponding author VCL upon reasonable request.
