# Supplementary material for: Impact of preoperative geriatric screening and comorbidity assessment in patients with vulvar and vaginal cancer
Source: J Cancer Res Clin Oncol. 2025 Dec 8;152(1):7. doi: 10.1007/s00432-025-06378-5 (PMC12686312; doi:10.1007/s00432-025-06378-5)
Supplement: Supplementary file 1 — Supplementary Material 1 [file 432_2025_6378_MOESM1_ESM.docx]

***Supplementary Table 1: Handgrip strength (since 2023, six patients)***

| **Handgrip strength**  (best of 3 trials) | **G8 negative**  **N= 5** | **G8 positive**  **N= 1** |
| --- | --- | --- |
| Right hand [kg] (+/- SD) (dominant) | 25.20 (+/- 7.0) | 16.0 |
| Left hand [kg] (+/- SD) | 22.80 (+/- 5.4) | 16.0 |

***Supplementary Table 2: Results of the second screening algorithm***

| **Test** | **G8 positive**  **N (%)** |
| --- | --- |
| History of falls in the last 6 months:  0  ≥ 1 | 6 (75)  2 (25) |
| Mini-Cog  Normal  Abnormal  Not performed | 3 (37.5)  4 (50.0)  1 (12.5) |
| Barthel Index  100 points: patient fully independent in activities of daily living  85–95 points: minor dependence; occasional assistance required  35–80 points: moderate dependence; regular assistance required  ≤ 30 points: severe dependence; patient largely care-dependent | 3 (37.5)  2 (25)  2 (25)  1 (12.5) |
| Geriatric Depression Scale  Normal  Abnormal | 5 (62.5)  3 (37.5) |
| Timed Up and Go (TUG)  Normal  Abnormal  Not performed | -  1 (12.5)  7 (87.5) |
